# Utility of a Modified Penlight‐Cover Test for Neurolocalization of Lesions Based on Visual Suppression of Nystagmus in Dogs and Cats With Vestibular Disease

**DOI:** 10.1111/jvim.70182

**Published:** 2025-06-27

**Authors:** Alice Chan, Gemma E. Longson, Edward Ives, Claire Turner, Paul Freeman, Stacey Brady, Ana Martinez Loro, Bruno Scalia, Susana Monforte Monteiro, Sara Formoso, Sam Khan, An E. Vanhaesebrouck

**Affiliations:** ^1^ Queen's Veterinary School Hospital University of Cambridge Cambridge UK; ^2^ Eastcott Veterinary Clinic and Hospital Swindon UK; ^3^ Anderson Moores Veterinary Specialists Winchester UK

**Keywords:** neurolocalisation, nystagmus, veterinary

## Abstract

**Background:**

Humans with peripheral vestibular disorders can suppress nystagmus through visual fixation, a capability often compromised in those with central vestibular disorders. Bedside tests that exploit this difference can aid neurolocalization in humans. These tests remain unexplored in veterinary medicine.

**Hypothesis:**

Removal of visual input will reveal or enhance nystagmus in animals with peripheral vestibular disease, while animals with central vestibular disease would show little change.

**Animals:**

Twenty‐one dogs and cats with peripheral vestibular lesions and 16 with central vestibular lesions. Diagnosis was confirmed by MRI.

**Methods:**

A prospective study was conducted using a modified penlight‐cover test. Because animals cannot be easily instructed to fixate on a visual target, removal of visual input was used as a substitute for eliminating visual fixation, based on the assumption that visual fixation also occurs spontaneously. A 0.5‐W LED penlight was shined into one eye while covering the other to eliminate visual input. Nystagmus beat frequency (BF) and subjective evaluation of slow phase velocity (SPV) were recorded before and during penlight application.

**Results:**

In animals with peripheral lesions, BF increased in 33% and SPV in 24% of cases after removal of visual input. Among those with central lesions, only one of 16 showed an increase in BF, and none exhibited an increase in SPV.

**Conclusions:**

When used alongside the neurological examination, the modified penlight‐cover test, could raise suspicion of a peripheral vestibular lesion if it reveals increased BF or SPV.

**Abbreviations**
BF: beat frequency
CSF: cerebrospinal fluid
CT: computed tomography
MRI: magnetic resonance imaging
PCT: penlight‐cover test
SPV: slow phase velocity

## 1 Introduction

Differentiation between peripheral and central vestibular neurolocalization is essential for guiding clinical management. While peripheral vestibular disorders often have no identifiable cause or arise from otitis or neuritis, central vestibular disorders might indicate serious intracranial disease and should be prioritized for urgent diagnostics and treatment.

Current neurolocalization methods include history, otoscopic, and neurological examination [1, 2]. Magnetic resonance imaging (MRI) is usually required for final diagnosis [3]. Although the neurological examination plays a key role in neurolocalization, approximately 20% of dogs with a suspected peripheral neurolocalization are subsequently found to have intracranial disease upon further imaging [3, 4].

Additional tools to support neurolocalization could help prioritize those requiring urgent imaging. In human medicine, the effect of visual fixation (the ability to maintain a steady gaze on a stationary point [5]) on nystagmus is used to aid neurolocalization [6, 7]. Visual fixation typically suppresses nystagmus of peripheral origin but has little to no effect on central nystagmus [6, 8, 9]. This response can be assessed using various techniques [6, 10, 11, 12], one being the penlight‐cover test **[** 13].

During the penlight‐cover test, the person is asked to fixate on a target [13]. A bright penlight is shone into one eye while the other is covered, removing visual fixation from both eyes and allowing observation of nystagmus in the uncovered eye [13]. In a human model of peripheral vestibulopathy, removal of visual fixation resulted in an increase in the slow phase velocity of nystagmus [13].

**FIGURE 1:**
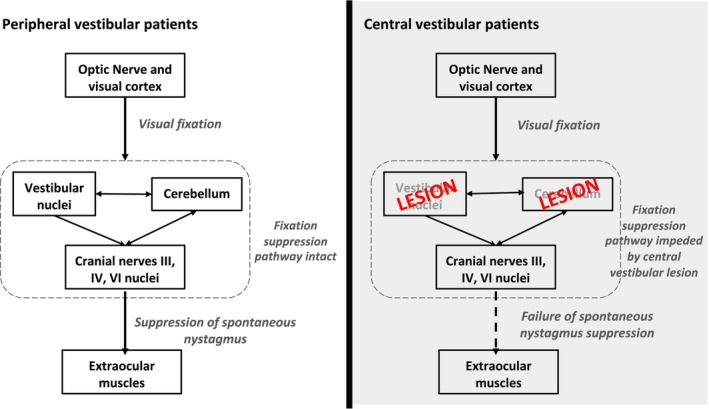
Schematic diagram to show how spontaneous nystagmus can be suppressed through visual fixation in peripheral vestibular disease as the fixation suppression pathway is intact; central vestibular disease might result in failure of spontaneous nystagmus suppression due to central vestibular lesions impeding the fixation suppression pathway. In this study, we used removal of visual input as a substitute for removal visual fixation, as it is difficult to instruct animals to fixate.

The visual fixation pathway involves complex integration of the visual system, vestibular nuclei, cerebellum (especially the flocculus and nodulus), and cranial nerves III, IV, and VI (Figure 1) [14, 15, 16]. This pathway enables humans and animals with peripheral vestibular dysfunction to suppress nystagmus. However, central lesions, particularly those involving the brainstem or cerebellum, typically impair this pathway, thereby abolishing the ability to suppress nystagmus through fixation [8, 17]. Exceptions exist, particularly in cases of focal cerebellar infarcts, where nystagmus suppression might still occur [18, 19]. Visual suppression of nystagmus has been documented in cats, monkeys, and humans [11, 20, 21], but not in dogs.

In this study, the penlight‐cover test is adapted for veterinary use, where instructed fixation is difficult to apply. Like the human version, it involves covering one eye and shining a bright light into the other tested eye, effectively dazzling the animal and preventing the animal from spontaneously fixating. Unlike the human test, it omits the instruction to fixate on a point. Previous studies have shown that eliminating visual input alone, without instructed fixation, can enhance nystagmus [11, 20, 22, 23]. This suggests that even in the absence of explicit instruction, spontaneous fixation is active and capable of suppressing nystagmus. Peripheral vestibular nystagmus in humans often goes undetected unless visual fixation is removed (e.g., with goggles) [6].

This prospective study aims to explore the modified penlight‐cover test in dogs and cats with vestibular signs. We hypothesized that animals with peripheral vestibular lesions would suppress spontaneous nystagmus through visual input, whereas those with central lesions would not. The aim was to assess whether the modified penlight‐cover test could serve as a complementary tool to the neurological examination for differentiating between peripheral and central vestibular neurolocalization, with definitive diagnosis determined by MRI.

## 2 Materials and Methods

This prospective study, approved by the University of Cambridge's Ethical Committee (CR473e), recruited dogs and cats presenting with vestibular signs from the Queen's Veterinary School Hospital and Anderson Moores Veterinary Specialists. Inclusion criteria included (1) the presence of vestibular ataxia, head tilt, positional strabismus, or jerk nystagmus at the time of presentation, (2) a neurological examination to determine neurolocalization, and (3) MRI of the brain and tympanic bullae. Further diagnostic tests, like cerebrospinal fluid analysis, infectious disease titres and myringotomy were performed as appropriate for each individual case. Animals with blindness and those ultimately diagnosed with lesions outside the vestibular system were excluded from the study. The penlight‐cover test was performed on the day of the animal's admission, by assessors who were blinded to the clinical history, neurological examination findings and final diagnosis.

**FIGURE 2:**
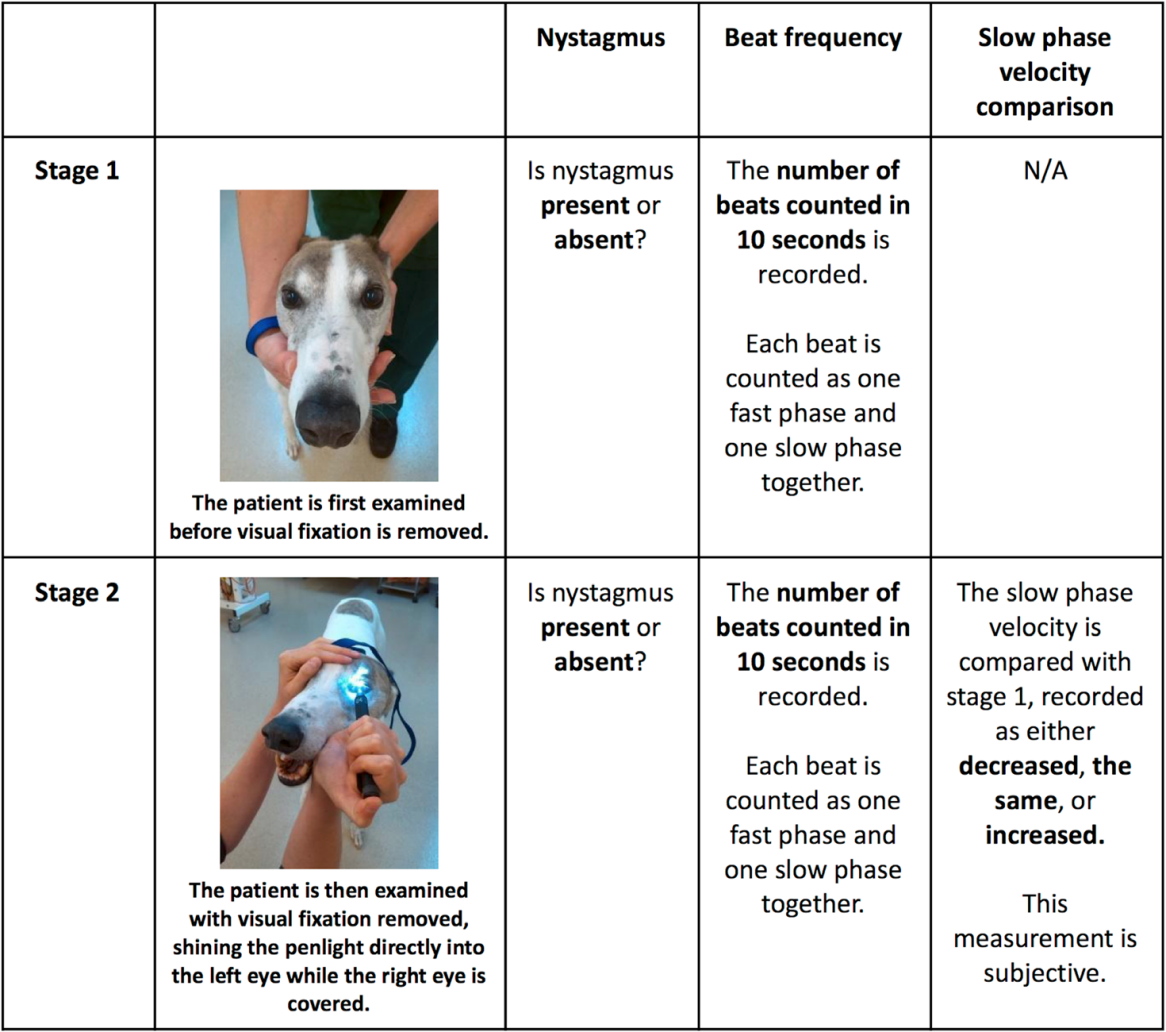
Modified penlight‐cover test protocol. Stage 1 is before visual input has been removed. Stage 2 is when visual input is removed. Characteristics of nystagmus are recorded as shown. Beat frequency was measured in 10‐s intervals. In the first 12 animals, it was measured over 7 s but extrapolated to 10 s for data analysis.

The penlight‐cover test was conducted in a well‐lit room, as follows (Figure 2): the animal was restrained with its head held straight and horizontal. A 0.5‐watt LED penlight (20 lm bulb) and a timer were used. Initially, the animal was examined before visual input was removed: the presence (or absence) of nystagmus and its beat frequency (i.e., number of fast phases per 10 s) were recorded. To remove visual input, the examiner shone the penlight directly into one eye (typically the left eye for right‐handed individuals) for 10 s while covering the other eye. During this time, nystagmus and its beat frequency were recorded again. The examiner also subjectively assessed whether the velocity of eye movements during the slow phase remained the same, increased, or decreased during penlight application.

For each animal, findings were later recorded in a standardized table, along with species, signalment, time from onset of vestibular signs to presentation, and neurological examination findings. Animals were categorized into peripheral or central (vestibular) groups, based on lesion localization and diagnosis determined by MRI. Animals with no lesion and suspected idiopathic vestibular syndrome were included in the peripheral group. Animals with lesions affecting both peripheral and central parts of the vestibular system, such as intracranial extension of otitis media‐interna, were included in the central group.

Fisher's exact test, chi‐squared test or *t*‐test was used to compare baseline characteristics between the peripheral and central groups. A Mann–Whitney *U* test was used to compare the change in beat frequency, while Fisher's exact test was used to compare the change in slow phase velocity between the groups. All tests were two‐tailed, unless a clear one‐tailed hypothesis was formulated a priori, as was the case for the beat frequency and slow phase velocity. SPSS (SPSS Inc., Chicago, IL 29.0) and JASP (JASP Team, 2024) statistical software were used for statistical testing and graphs. A *p*‐value of less than 0.05 was considered statistically significant. To assess the utility of the test, likelihood ratios, their 95% confidence intervals (CIs) and post‐test probabilities were calculated and a Fagan's nomogram graphed, using an online calculator (Schwartz A, diagnostic test calculator, 2001). A positive test result was defined as an increase in nystagmus beat frequency during the modified penlight‐cover test compared to baseline. A negative test result was defined as no increase in beat frequency.

## 3 Results

### 3.1 Sample Characteristics

**FIGURE 3:**
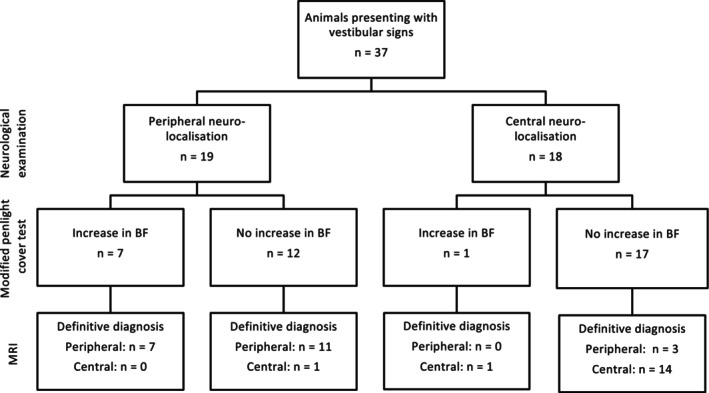
Flow chart illustrating an overview of the clinical reasoning process for animals, including neurolocalization after neurological examination, modified penlight‐cover test assessment, and diagnosis after MRI. BF, beat frequency.

A total of 37 animals met the inclusion criteria with 21 in the peripheral group and 16 in the central group. Demographic data of both groups is in Table 1. Baseline characteristics, such as age at onset and duration of signs before presentation, were similar between the two groups. A flowchart illustrating the test outcomes, including neurolocalization based on neurological examination, modified penlight‐cover test results, and final diagnosis based on MRI findings, is presented in Figure 3.

**TABLE 1:** Demographic characteristics of animals between peripheral and central vestibular groups.

	MRI‐confirmed lesion localization and diagnosis	
	Peripheral vestibular group (*n* = 21)^**a**^	Central vestibular group (*n* = 16)
Dog, *n* (%)	15 (71%)	14 (88%)
Cat, *n* (%)	6 (29%)	2 (12%)
Male, *n* (%)	12 (57%)	10 (62%)
Female, *n* (%)	9 (43%)	6 (38%)
Age (months)^b^	96 (57.5–154.5)	56 (37.5–104.5)
Time since vestibular signs were first noticed (hours)^b^	96 (24–414)	108 (24–480)

*Note:* *n*, number.

^a^ Includes idiopathic vestibular syndrome (*n* = 10).

^b^ Median (interquartile range).

### 3.2 Change in Beat Frequency

**FIGURE 4:**
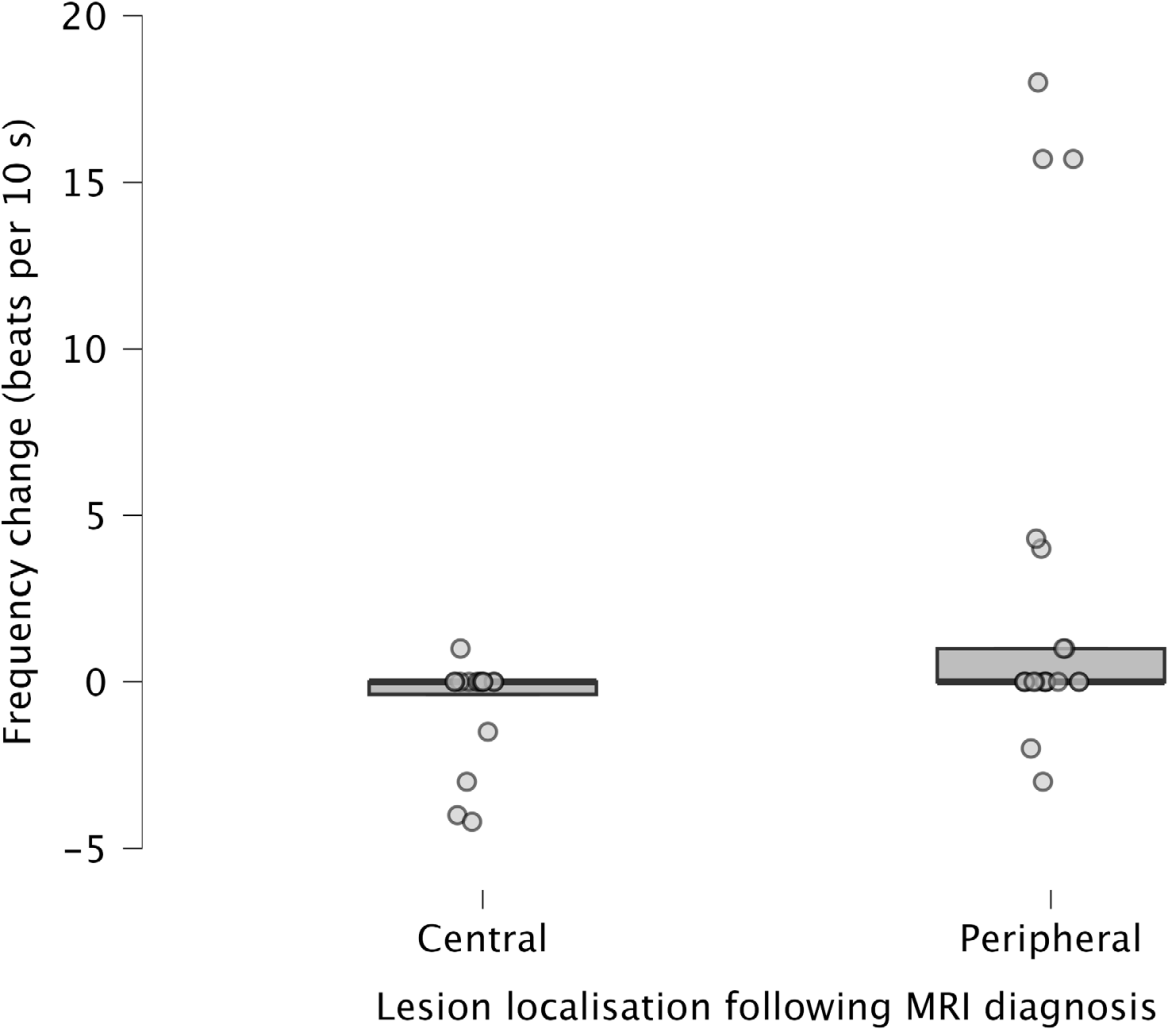
Box plot to show the change in beat frequency in 21 animals with peripheral lesion (median: 0; interquartile range, IQR: 0–1) and 16 animals with central lesion (median: 0; IQR: −0.375 to 0). *p* = 0.015, one‐tailed Mann–Whitney *U* test.

Before visual input was removed, spontaneous nystagmus was present in nearly half of the animals (48% [*n* = 10/21] for the peripheral group, 44% [*n* = 7/16] for the central group). For each animal, the change in beat frequency before and after the removal of visual input was calculated. An increase in beat frequency occurred in a third of animals of the peripheral group (33% [*n* = 7/21]) and only in one animal of the central group (6% [*n* = 1/16], +1 beat/10 s). The change in beat frequency differed significantly between the peripheral and central groups (*p* = 0.015; Figure 4). Due to uncertainty regarding lesion origin in idiopathic cases (*n* = 10) and cases with both peripheral and central lesions (*n* = 1), the analysis was repeated after excluding these cases. The difference remained significant (*p* = 0.004).

### 3.3 Change in Slow Phase Velocity

Nearly a quarter of animals in the peripheral group (24% [*n* = 5/21]; 95% CI: 10.6–45.1) showed an increase in slow phase velocity when visual input was removed. One of these five animals had no spontaneous nystagmus before the test but developed it during testing. None of the animals in the central group (0% [*n* = 0/16]; 95% CI: 0–19.4) showed an increase in slow phase velocity when visual input was removed. An increased slow phase velocity was significantly associated with a peripheral lesion diagnosis (Fisher exact test, *p* = 0.047), and this association remained significant after excluding idiopathic cases and the single case with concurrent peripheral and central lesions (*p* = 0.022).

### 3.4 Likelihood Ratios and Bayesian Reasoning

**FIGURE 5:**
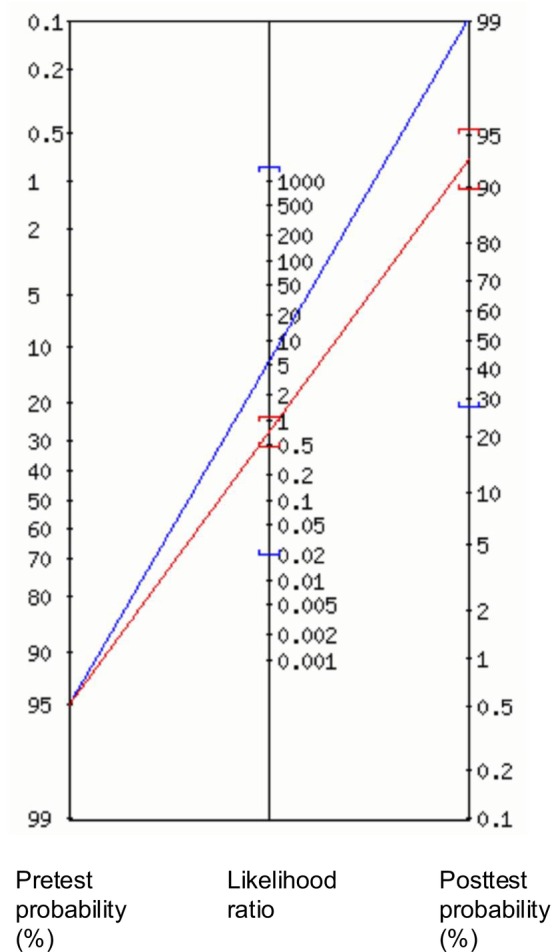
A Fagan's nomogram demonstrating the application of the positive (blue line) and negative (red line) likelihood ratios to animals initially localized peripherally. This figure was generated by the online diagnostic test calculator, Alan Schwartz, 2002.

Bayesian inference, using likelihood ratios, assesses the clinical utility of a test or series of tests. In this study, the series includes the neurological examination followed by the modified penlight‐cover test. It addresses the question “what is the probability of having the disease, or more specifically relevant to our work, a lesion in this location, given the successive test results” [24]. This effect is visualized in a Fagan's nomogram (Figure 5). For a full explanation the reader is directed to Gill et al. [24].

In our study, a positive result (increased beat frequency) was obtained in 33% of peripheral cases and 6% of central cases, giving a positive and negative likelihood ratio of 5.3 (95% CI: 0.73–39.0) and 0.71 (95% CI: 0.51–0.99), respectively. For animals with a peripheral neurolocalization based on neurological examination alone, the pretest probability of peripheral vestibular disease was 95% (calculated from Figure 3). Applying the likelihood ratios, a positive modified penlight‐cover test increases this probability to 99% (with a 1% chance of central disease), while a negative test reduces it to 93% (Figure 5). The likelihood ratios can also be applied to animals initially assigned a central neurolocalization based on neurological examination, with a pretest probability of peripheral disease of 17%. An increase in beat frequency increases this to 52%, while no change decreases the probability to 13%.

More details, including nystagmus characteristics, MRI diagnosis, and results of repeated testing of the modified penlight‐cover test in some dogs, are in Table S1.

## 4 Discussion

This study investigated how removing visual input (as a substitute for eliminating visual fixation) alters spontaneous pathological nystagmus in dogs and cats. Covering one eye while shining a bright penlight into other, thereby removing visual input, increased nystagmus beat frequency and slow phase velocity more often in animals with peripheral lesions than in those with central lesions. This supports the hypothesis that peripheral vestibular lesions permit visual suppression of nystagmus, where central lesions might impair the neural pathways involved in this suppression.

Most animals in the peripheral group did not show an increase in beat frequency (67%) or slow phase velocity (76%). Similarly, a human study found no significant increase in 65% of patients with peripheral vestibular disease using the penlight‐cover test [13]. Several factors could limit the test's sensitivity. Discrete central nervous system lesions (e.g., transient ischemic attacks) could have been missed on MRI, which is particularly relevant to idiopathic vestibular syndrome. Eight of the 10 dogs with idiopathic vestibular syndrome in our cohort showed no increase in beat frequency or slow‐phase velocity, and one also showed central signs despite a normal MRI. Although typically diagnosed by excluding MRI‐visible lesions and categorized as a peripheral disease, it is uncertain whether idiopathic vestibular syndrome is exclusively peripheral. Secondly, the test's low sensitivity might stem from the method itself. In humans, video Frenzel and infrared goggles are now commonly used to eliminate visual fixation, enabling more sensitive and accurate assessments of nystagmus [10]. Variations in room lighting might also influence results, as brighter lighting enhances visual suppression of nystagmus [11]. Additionally, some studies use caloric or rotational stimulation to elicit nystagmus [13, 25], whereas our study relied on spontaneous pathological nystagmus, which was not consistently present at baseline. Finally, blocking visual input might not fully replicate removal of true visual fixation, potentially limiting the assessment of both suppression and reinstatement of nystagmus.

As nystagmus can be subtle, other methods that reduce visual input, such as using an ophthalmoscope or magnifying lens in a dim or dark room [10, 11], could improve nystagmus detection and improve the sensitivity of the proposed test. Repeating the modified penlight‐cover test multiple times in each animal could also improve its sensitivity. For instance, in two dogs from the peripheral group, the appearance of nystagmus or a rise in beat frequency was detected during repeated testing that was not evident initially (Table S1).

We used Bayesian inference to demonstrate that an increase in nystagmus beat frequency, when combined with neurological examination, can increase confidence in vestibular neurolocalization. The modified penlight‐cover test is most useful when the pretest probability of peripheral vestibular disease is low. The pretest probability refers to the likelihood of a final peripheral diagnosis in animals with a peripheral vestibular neurolocalization based solely on neurological examination. In our study, the pretest probability was already very high (95%), similar to other veterinary studies [26, 27]. An increase in beat frequency using the modified penlight‐cover test further increased this probability to 99% (Figure 5). Other studies showed a lower pre‐test probability (77%–81%) [3, 4]. In these situations, and using our data, an increase in beat frequency using the modified penlight‐cover test would increase the probability of a peripheral diagnosis more significantly (from 80% to 95%), potentially guiding clinical decision‐making more substantially.

We only examined one eye for nystagmus changes, while covering the other, and did not repeat the test on the opposite side. This is unlikely to have affected the results, as vestibular nystagmus is typically conjugate, affecting both eyes. We also acknowledge that maintaining a straight, horizontal head position can be challenging, especially in dogs with severe vestibular dysfunction. This position is crucial to prevent positional nystagmus.

In conclusion, increases in beat frequency and slow phase velocity during the modified penlight‐cover test were more common in dogs and cats with peripheral vestibular lesions than in those with central lesions diagnosed by MRI. These increases can therefore corroborate the suspicion of a peripheral neurolocalization, though their absence is not informative. The modified penlight‐cover test should be used as a supplementary tool to the neurological examination, aiding in neurolocalization, but should not replace it or serve as a definitive diagnostic tool.

## Disclosure

Off‐label antimicrobials were not administered as part of study methodology but might have been prescribed by the clinician on a case‐by‐case basis.

## Ethics Statement

Ethical approval by the University of Cambridge, CR473. Authors declare human ethics approval was not needed.

## Conflicts of Interest

The authors declare no conflicts of interest.

## Supporting information


**Table S1.** Clinical data of animals enrolled in the study.
